# Social norms towards smoking and vaping and associations with product use among youth in England, Canada, and the US

**DOI:** 10.1016/j.drugalcdep.2019.107635

**Published:** 2019-12-01

**Authors:** Katherine A. East, Sara C. Hitchman, Ann McNeill, James F. Thrasher, David Hammond

**Affiliations:** a National Addiction Centre, Institute of Psychiatry, Psychology and Neuroscience, King’s College London, UK; b UK Centre for Tobacco and Alcohol Studies, UK; c Department of Health Promotion, Education, and Behavior, Arnold School of Public Health, University of South Carolina, USA; d Department of Tobacco Research, Center for Population Health Research, National Institute of Public Health, Mexico; e School of Public Health and Health Systems, University of Waterloo, Canada

**Keywords:** Electronic cigarettes, Vaping, Smoking, Social norms, Youth, Survey

## Abstract

**Background:**

This study assesses differences in social norms towards smoking and vaping among youth across countries (England, Canada, US) and smoking and vaping status.

**Methods:**

Data are from the 2017 ITC Youth Tobacco and Vaping Survey, among youth age 16–19 in England (N = 3444), Canada (N = 3327), and US (N = 3509). Prevalence of friend smoking, friend vaping, peer approval of smoking, and peer approval of vaping were estimated. Adjusted logistic regression models were estimated for each norm to assess associations with country, smoking status, and vaping status, adjusting for sociodemographics, alcohol use, and marijuana use.

**Results:**

47% and 52% reported friend smoking and vaping respectively. Perceived peer approval of vaping (44%) was almost double that of smoking (23%). Compared with England, fewer Canadian and US youth reported friend smoking (Canada: AOR = 0.71 [95% CI = 0.62–0.82]; US: AOR = 0.54 [0.47–0.62]) and peer approval of smoking (Canada: AOR = 0.74 [0.63–0.87]; US: AOR = 0.78 [0.67–0.91]), yet more reported peer approval of vaping (Canada: AOR = 1.23 [1.08–1.41]; US: AOR = 1.30 [1.14–1.48]). More Canadian than English youth reported friend vaping (AOR = 1.17 [1.02–1.36]). Friend smoking, peer approval of smoking, and friend vaping were more common among smokers and vapers (all p < .02). Peer approval of vaping was more common among vapers but less common among smokers (all p < .044).

**Conclusions:**

Youth had more positive vaping than smoking norms. English youth reported the most pro-smoking but least pro-vaping norms in adjusted models; this was unexpected given country differences in regulatory environments. Norms towards both products were associated with use, with some evidence of cross-product associations between norms and behaviours.

## Highlights

• England had more pro-smoking but less pro-vaping norms overall.
• Canada and US differed on friend smoking only, which was greater in Canada than US.
• Country differences cannot easily be explained by prevalence or policies.
• Smokers had more pro-smoking norms, vapers had more pro-vaping norms.
• There were also some cross-product associations between norms and product use.

## 1 Introduction

Tobacco smoking is the leading preventable cause of death and disease worldwide, killing over seven million people annually (World Health Organization [WHO], 2018). In many countries, increasingly comprehensive tobacco control policies have been implemented with the aim of “denormalizing” smoking and reducing smoking prevalence (Chapman and Freeman, 2008; Dubray et al., 2015). However, the nicotine market has changed with the introduction of vaping devices (also called e-cigarettes; Hon, 2003) and there has been considerable discussion with regards to the impact of vaping on smoking norms and behavior. Vaping devices have the potential to reduce the harms caused by smoking and may help some smokers quit (Hajek et al., 2019; McNeill et al., 2019). However, concerns have been expressed that e-cigarettes might “renormalize” and promote smoking, particularly among youth (Aveyard et al., 2018; Sæbø and Scheffels, 2017; The European Parliament and the Council of the European Union, 2014; US Department of Health and Human Services, 2016). Studies are therefore needed to assess social norms towards smoking and vaping among youth, particularly cross-product associations between norms and behavior.

Social norms can be classified into two domains: descriptive and injunctive. Descriptive norms refer to perceptions of how others behave (e.g., friend smoking), while injunctive norms refer to perceptions of what others think people should or should not do (e.g., peer approval of smoking) (Borsari and Carey, 2003; Cialdini et al., 1991). Associations between descriptive and injunctive norms and youth smoking are well documented: youth who have more friends or peers who smoke (Chang et al., 2006; Conner et al., 2017; Lotrean et al., 2013) or who perceive greater approval of smoking among parents, friends, or peers (Chang et al., 2006; Lotrean et al., 2013; Van De Ven et al., 2007) are more likely to initiate smoking. Moreover, cross-sectional studies in the US and Mexico have found that youth with friends who vape and who perceive acceptability of vaping among peers are more susceptible to (i.e., open to trying in the next year), and more likely to actually try, vaping (Gorukanti et al., 2017; Lozano et al., 2019; Thrasher et al., 2016).

It is less clear whether there are cross-product influences of norms on product use. In Mexico and Argentina, friend smoking was more common among youth who have tried and are susceptible to vaping (Lozano et al., 2019; Morello et al., 2016; Thrasher et al., 2016); however, no association was found between friend vaping, or perceived social acceptability of vaping, and smoking susceptibility (Lozano et al., 2019). By contrast, a longitudinal study in Britain found that youth with vaping friends had greater odds of initiating vaping but lower odds of initiating smoking, while those who perceived public approval of smoking had lower odds of initiating vaping (East et al., 2018). It is therefore possible that norms towards one product may suppress, rather than promote, use of the alternative. Further research is required to corroborate these findings, particularly across countries with different smoking and vaping environments.

Research suggests that adult smokers and ex-smokers from countries with stronger tobacco control policies have more anti-smoking injunctive norms (Hammond et al., 2006; Kasza et al., 2017), while those from countries with less restrictive vaping policies have more pro-vaping norms (Aleyan et al., 2019). By contrast, a recent survey of smokers in Europe (East et al., 2019a) found that while friend smoking was more common in countries with greater smoking prevalence and weaker tobacco control policies, approval of smoking was not, and friend vaping and approval of vaping showed little obvious relation to vaping prevalence or policies. However, adult smokers and ex-smokers likely have unique norms towards smoking, and there is no research of which we are aware comparing smoking and vaping norms across countries among youth.

This study therefore assesses whether social norms towards smoking and vaping are associated with country, smoking status, and vaping status among 16–19-year-olds in Canada, England, and the US. At the time of surveying, prevalence of ever smoking and ever vaping among 16–19-year-olds were lowest in Canada but similar in England and the US (Hammond et al., 2019). England and Canada had more comprehensive tobacco control policies than the US, having implemented comprehensive smoke free legislation, retail display bans, bans on tobacco advertising, and mandated graphic health warnings on cigarette packs (ITC, 2018; WHO, 2017). Canada had more restrictive policies on the sale, use, and advertisement of vaping devices than England and the US (Gravely et al., 2019; ITC, 2018), although these were generally unenforced (Hammond et al., 2015).

## 2 Methods

### 2.1 Design

A full description of the methods can be found in Hammond et al. (2018). Briefly, data were from Wave 1 (July/August 2017) of the International Tobacco Control Policy Evaluation Project (ITC) Youth Tobacco and Vaping Survey, an online survey of 16–19-year-olds in England, Canada, and US. Respondents were recruited through Nielsen Consumer Insights Global Panel (and partners’ panels) directly or through their parents. Email invitations were sent to a random sample of panelists after targeting for age criteria. Panelists not aged 16–19, had no children aged 16–19, and/or were attempting to complete the survey on a mobile device were ineligible to partake. The survey was in English, and French in Canada, and took approximately 15 minutes. The same measures were used in all countries except ethnicity, region, and education, which were based on country census questions. Informed consent was required, and respondents received remuneration according to their panel’s incentive structure. Ethical clearance was received from the University of Waterloo Research Ethics Committee (AORE#21847) and King’s College London’s Psychiatry, Nursing & Midwifery Research Ethics Subcommittee (PNM-RESC-HR-16/17-4113).

### 2.2 Sample

The survey was completed by 13,468 youth age 16–19-years, of which 10,280 were retained for this study. The following were excluded: those who provided incomplete/invalid data on smoking/vaping status or other variables used for weighting (n = 1120), failed data quality checks (n = 382), did not report or responded “Don’t know” to friend smoking (n = 343), friend vaping (n = 309), peer approval of smoking (n = 316), peer approval of vaping (n = 718).

### 2.3 Measures

#### 2.3.1 Social norms (outcomes)

(i) *Friend smoking*: “Who, if anyone… smokes cigarettes?”, followed by a list of people. Respondents who checked “Your friend(s)” were coded as having friends who smoke.
(ii) *Friend vaping*: “Who, if anyone… uses e-cigarettes/vapes?”, followed by a list of people. Respondents who checked “Your friend(s)” were coded as having friends who vape.
(iii) *Peer approval of smoking*: “Do people your age approve or disapprove of smoking cigarettes?” (a) Strongly approve, (b) Somewhat approve, (c) Neither approve nor disapprove, (d) Somewhat disapprove, (e) Strongly disapprove. (a)-(b) were coded as approve; (c)-(e) were coded as not approve.
(iv) *Peer approval of vaping*: “Do people your age approve or disapprove of using e-cigarettes/vaping?” (a) Strongly approve, (b) Somewhat approve, (c) Neither approve nor disapprove, (d) Somewhat disapprove, (e) Strongly disapprove. (a)-(b) were coded as approve; (c)-(e) were coded as not approve.

Coding of (iii) and (iv) is consistent with similar studies (Lozano et al., 2019; East et al., 2019a).

#### 2.3.2 Smoking and vaping status

*Smoking status*: Current (smoked 100+ cigarettes in life and smoked in past-30-days), experimental (tried smoking, but did not smoke 100+ cigarettes in life), former (smoked 100+ cigarettes in life, but did not smoke in past-30-days), never (never tried smoking, not even a puff) (Hammond et al., 2018).

*Vaping status*: Current (vaped 100+ days in life and vaped in past-30-days), experimental (tried vaping, but did not vape 100+ days in life), former (vaped 100+ days in life, but did not vape in past-30-days), never (never tried vaping, not even a puff) (Hammond et al., 2018).

#### 2.3.3 Covariates

Covariates included age (16–19), sex (male, female), ethnicity (white, other/mixed, don’t know/refused), current student (yes, no, don’t know/refused), monthly alcohol use (yes, no, don’t know/refused), past-30-day marijuana use (yes, no, don’t know/refused), and two socio-economic status indicators: number of computers in household (0–2, ≥3, don’t know/refused), number of bathrooms in household (0–1, ≥2, don’t know/refused) (Hartley et al., 2013).

### 2.4 Analyses

All analyses were conducted using Stata v15. First, sample characteristics were examined in each country. Second, prevalence of each social norm was estimated. Third, four separate adjusted logistic regression models were estimated for each social norm to assess associations with country, smoking status, vaping status, and covariates. Fourth, interactions between country and smoking status, and country and vaping status, were added as separate additional steps to the fully-adjusted models. For interactions, experimental and former smokers, and experimental and former vapers, were combined due to low numbers of former smokers and former vapers. Adjusted Wald tests were performed on the interaction terms following model specification; where there was evidence for an interaction (p ≤ .05) average predicted probabilities and pairwise comparisons were generated using Stata’s margins command. All analyses use weighted data unless otherwise indicated, with sample weights constructed based on smoking status, region, language (Canada), sex, age, ethnicity, and using a raking algorithm described in Hammond et al. (2018).

## 3 Results

### 3.1 Sample characteristics

Most participants were 18, male, white, students, did not use alcohol monthly (except England), did not use marijuana in the past-30-days, had ≥3 computers and ≥2 bathrooms (except England) in household, and had never smoked or vaped (Table 1).

**Table 1:** Sample characteristics by country.

	Unweighted n (weighted %)		
	England (N = 3444)	Canada (N = 3327)	US (n = 3509)
**Age**			
16	512 (18.82)	503 (19.15)	736 (22.29)
17	856 (29.80)	775 (27.30)	777 (23.54)
18	1226 (30.04)	1087 (29.95)	1105 (30.48)
19	850 (21.34)	962 (23.59)	891 (23.69)
**Sex**			
Male	1456 (54.96)	1132 (50.90)	1376 (53.40)
Female	1988 (45.04)	2195 (49.10)	2133 (46.60)
**Ethnicity**			
White	2720 (79.57)	1818 (59.46)	2322 (74.02)
Other/mixed	696 (19.58)	1459 (39.22)	1171 (25.54)
Don’t know/refused	28 (0.84)	50 (1.32)	16 (0.44)
**Student**			
Yes	3124 (89.81)	3080 (89.76)	3121 (86.79)
No	298 (9.42)	239 (9.95)	380 (13.09)
Don’t know/refused	22 (0.77)	8 (0.29)	8 (0.12)
**Monthly alcohol use**			
No	1462 (44.64)	2011 (63.35)	2700 (76.36)
Yes	1934 (53.93)	1254 (34.84)	740 (21.55)
Don’t know/refused	48 (1.43)	62 (1.81)	69 (2.10)
**Past-30-day marijuana use**			
No	3088 (88.00)	2860 (85.64)	2994 (83.05)
Yes	298 (10.16)	416 (12.94)	445 (14.46)
Don’t know/refused	58 (1.83)	51 (1.42)	70 (2.49)
**Computers in household**			
0-2	684 (21.58)	640 (22.18)	960 (30.01)
≥3	2701 (76.73)	2644 (76.50)	2524 (69.20)
DK/refused	59 (1.70)	43 (1.31)	25 (0.78)
**Bathrooms in household**			
0-1	1753 (53.19)	800 (27.73)	728 (20.69)
≥2	1671 (46.20)	2503 (71.45)	2771 (79.01)
DK/refused	20 (0.61)	24 (0.82)	10 (0.30)
**Smoking status**			
Never	2000 (61.76)	2307 (77.63)	2317 (58.49)
Former	26 (1.74)	21 (1.63)	23 (1.46)
Experimental	1214 (21.77)	857 (8.89)	1007 (29.24)
Current	204 (14.72)	142 (11.85)	162 (10.80)
**Vaping status**			
Never	2266 (64.36)	2351 (72.20)	2311 (61.72)
Former	17 (0.96)	12 (0.72)	27 (1.15)
Experimental	1115 (31.83)	905 (24.03)	1084 (32.56)
Current	46 (2.84)	59 (3.04)	87 (4.57)

### 3.2 Prevalence of each social norm

Across all three countries, 46.7% and 51.6% had friends who smoked and vaped respectively. Peer approval of smoking (23.1%) was just over half that of peer approval of vaping (44.3%). Prevalence of smoking and vaping norms in each country is shown in Table 2, Table 3, respectively.

**Table 2:** Adjusted associations between youth reporting that their friends smoke and their friends vape (i.e., descriptive norms) and: country, smoking status, vaping status, and all covariates (n = 10,280). All data are weighted unless otherwise stated.

	Unweighted n (% of full sample)	Friends smoke (vs. otherwise)			Friends vape (vs. otherwise)		
		%	AOR (95% CI)	p	%	AOR (95% CI)	p
**Country**							
England (ref)	3444 (33.1)	56.2	1.00		51.6	1.00	
Canada	3327 (32.3)	43.2	**0.71 (0.62-0.82)**	**<.001**	50.4	**1.17 (1.02-1.36)**	**.029**
US	3509 (34.6)	40.8	**0.54 (0.47-0.62)**	**<.001**	52.8	1.10 (0.96-1.27)	.162
**Smoking status**							
Never (ref)	6624 (65.8)	33.2	1.00		40.4	1.00	
Former	70 (1.6)	79.5	**5.25 (2.57-10.72)**	**<.001**	85.1	1.50 (0.68-3.33)	.315
Experimental	3078 (20.2)	63.6	**2.62 (2.29-2.99)**	**<.001**	68.5	**1.27 (1.10-1.45)**	**.001**
Current	508 (12.4)	86.3	**8.10 (5.76-11.4)**	**<.001**	79.2	1.21 (0.89-1.64)	.226
**Vaping status**							
Never (ref)	6928 (66.0)	35.6	1.00		35.5	1.00	
Former	56 (1.0)	78.8	1.68 (0.86-3.26)	.128	87.4	**8.47 (2.67-26.93)**	**<.001**
Experimental	3104 (29.6)	67.0	**1.53 (1.34-1.76)**	**<.001**	81.4	**6.18 (5.32-7.18)**	**<.001**
Current	192 (3.5)	75.7	1.00 (0.63-1.57)	.996	95.6	**26.54 (10.59-66.51)**	**<.001**
**Age**							
16 (ref)	1751 (20.1)	38.3	1.00		42.6	1.00	
17	2408 (26.8)	42.2	1.06 (0.91-1.25)	.453	47.0	**1.18 (1.01-1.38)**	**.037**
18	3418 (30.2)	48.5	1.14 (0.98-1.33)	.081	55.0	**1.33 (1.14-1.54)**	**<.001**
19	2703 (22.9)	57.0	**1.37 (1.17-1.62)**	**<.001**	60.7	**1.44 (1.21-1.70)**	**<.001**
**Sex**							
Male (ref)	3964 (53.1)	47.5	1.00		53.0	1.00	
Female	6316 (46.9)	45.8	1.05 (0.95-1.17)	.307	50.1	0.97 (0.87-1.07)	.505
**Ethnicity**							
White (ref)	6860 (71.2)	47.9	1.00		51.5	1.00	
Other/mixed	3326 (28.0)	43.9	**1.13 (1.01-1.28)**	**.041**	52.3	**1.24 (1.10-1.39)**	**<.001**
Don’t know/refused	94 (0.9)	35.0	0.86 (0.50-1.48)	.584	42.6	0.96 (0.55-1.67)	.874
**Student**							
Yes (ref)	9325 (88.8)	45.6	1.00		50.5	1.00	
No	917 (10.9)	56.7	0.87 (0.71-1.05)	.145	60.6	0.90 (0.73-1.11)	.313
Don’t know/refused	38 (0.4)	24.1	**0.18 (0.05-0.69)**	**.013**	54.0	1.02 (0.45-2.30)	.971
**Monthly alcohol use**							
No (ref)	6173 (61.7)	36.2	1.00		43.9	1.00	
Yes	3928 (36.6)	64.4	**1.76 (1.56-1.98)**	**<.001**	64.4	**1.43 (1.26-1.62)**	**<.001**
Don’t know/refused	179 (1.8)	44.7	1.08 (0.71-1.64)	.724	59.8	1.43 (0.91-2.25)	.119
**Past-30-day marijuana use**							
No (ref)	8942 (85.5)	42.2	1.00		47.1	1.00	
Yes	1159 (12.6)	74.6	**1.41 (1.15-1.73)**	**.001**	79.7	**1.27 (1.01-1.60)**	**.045**
Don’t know/refused	179 (1.9)	65.7	1.48 (1.00-2.21)	.051	72.5	1.31 (0.89-1.93)	.168
**Computers in household**							
0-2 (ref)	2284 (24.7)	50.5	1.00		55.8	1.00	
≥3	7869 (74.1)	45.4	1.04 (0.91-1.18)	.579	50.4	1.01 (0.89-1.15)	.879
Don’t know/refused	127 (1.3)	45.1	1.15 (0.70-1.89)	.590	45.0	0.89 (0.55-1.44)	.643
**Bathrooms in household**							
0-1 (ref)	3281 (33.7)	52.0	1.00		53.9	1.00	
≥2	6945 (65.7)	44.0	0.99 (0.88-1.12)	.929	50.5	0.97 (0.86-1.10)	.614
Don’t know/refused	54 (0.6)	40.8	1.06 (0.49-2.32)	.877	46.5	1.28 (0.61-2.68)	.506

AOR = Adjusted Odds Ratio. 95% CI = 95% Confidence Interval.

**Table 3:** Adjusted associations between youth perceiving that their peers approve of smoking and their peers approve of vaping (i.e., injunctive norms) and: country, smoking status, vaping status, and all covariates (n = 10,280). All data are weighted unless otherwise stated.

	Unweighted n (% of full sample)	Peers approve of smoking (vs. not approve)			Peers approve of vaping (vs. not approve)		
		%	AOR (95% CI)	p	%	AOR (95% CI)	p
**Country**							
England (ref)	3444 (33.1)	25.4	1.00		40.2	1.00	
Canada	3327 (32.3)	21.1	**0.74 (0.63-0.87)**	**<.001**	45.6	**1.23 (1.08-1.41)**	**.002**
US	3509 (34.6)	22.7	**0.78 (0.67-0.91)**	**.002**	47.1	**1.30 (1.14-1.48)**	**<.001**
**Smoking status**							
Never (ref)	6624 (65.8)	19.7	1.00		42.7	1.00	
Former	70 (1.6)	28.7	1.14 (0.61-2.14)	.683	55.1	0.81 (0.47-1.40)	.457
Experimental	3078 (20.2)	27.3	**1.20 (1.03-1.39)**	**.019**	47.4	**0.82 (0.72-0.93)**	**.003**
Current	508 (12.4)	33.8	**1.44 (1.09-1.90)**	**.011**	46.5	**0.62 (0.48-0.79)**	**<.001**
**Vaping status**							
Never (ref)	6928 (66.0)	19.1	1.00		39.3	1.00	
Former	56 (1.0)	34.4	1.84 (0.91-3.72)	.088	47.1	**1.98 (1.02-3.83)**	**.044**
Experimental	3104 (29.6)	30.4	**1.57 (1.34-1.83)**	**<.001**	53.2	**2.08 (1.82-2.37)**	**<.001**
Current	192 (3.5)	34.6	**1.81 (1.16-2.81)**	**.008**	63.8	**3.86 (2.53-5.88)**	**<.001**
**Age**							
16 (ref)	1751 (20.1)	22.9	1.00		41.7	1.00	
17	2408 (26.8)	23.4	0.98 (0.82-1.16)	.784	41.2	0.97 (0.84-1.12)	.685
18	3418 (30.2)	22.4	**0.83 (0.70-0.99)**	**.036**	46.2	1.11 (0.96-1.28)	.152
19	2703 (22.9)	23.9	0.84 (0.70-1.02)	.074	47.8	1.13 (0.97-1.31)	.131
**Sex**							
Male (ref)	3964 (53.1)	21.7	1.00		40.9	1.00	
Female	6316 (46.9)	24.7	**1.27 (1.13-1.43)**	**<.001**	48.2	**1.37 (1.24-1.51)**	**<.001**
**Ethnicity**							
White (ref)	6860 (71.2)	21.2	1.00		42.5	1.00	
Other/mixed	3326 (28.0)	27.9	**1.61 (1.41-1.83)**	**<.001**	49.4	**1.30 (1.16-1.45)**	**<.001**
Don’t know/refused	94 (0.9)	23.2	1.23 (0.66-2.27)	.511	33.6	0.77 (0.45-1.32)	.350
**Student**							
Yes (ref)	9325 (88.8)	22.5	1.00		44.0	1.00	
No	917 (10.9)	28.2	1.21 (0.98-1.49)	.075	47.6	1.07 (0.89-1.28)	.460
Don’t know/refused	38 (0.4)	29.4	1.15 (0.48-2.77)	.752	33.2	0.74 (0.34-1.62)	.447
**Monthly alcohol use**							
No (ref)	6173 (61.7)	21.8	1.00		43.8	1.00	
Yes	3928 (36.6)	24.6	0.95 (0.82-1.10)	.493	45.3	0.98 (0.87-1.10)	.712
Don’t know/refused	179 (1.8)	36.1	**1.75 (1.18-2.59)**	**.005**	40.0	0.80 (0.54-1.19)	.276
**Past-30-day marijuana use**							
No (ref)	8942 (85.5)	21.6	1.00		43.0	1.00	
Yes	1159 (12.6)	32.8	**1.24 (1.00-1.52)**	**.047**	53.0	1.11 (0.92-1.34)	.274
Don’t know/refused	179 (1.9)	27.4	0.94 (0.59-1.48)	.776	44.9	0.90 (0.59-1.36)	.604
**Computers in household**							
0-2 (ref)	2284 (24.7)	27.0	1.00		45.5	1.00	
≥3	7869 (74.1)	21.8	**0.85 (0.74-0.98)**	**.026**	44.0	1.00 (0.89-1.14)	.964
Don’t know/refused	127 (1.3)	26.5	1.09 (0.61-1.94)	.766	40.8	1.12 (0.68-1.85)	.662
**Bathrooms in household**							
0-1 (ref)	3281 (33.7)	25.2	1.00		45.6	1.00	
≥2	6945 (65.7)	22.0	0.98 (0.86-1.13)	.823	43.7	**0.87 (0.78-0.97)**	**.016**
Don’t know/refused	54 (0.6)	25.7	0.92 (0.35-2.42)	.873	34.8	0.74 (0.34-1.61)	.452

AOR = Adjusted Odds Ratio. 95% CI = 95% Confidence Interval.

### 3.3 Associations between each social norm and country, smoking status, vaping status, and covariates

#### 3.3.1 Friend smoking

Respondents had greater odds of reporting that their friends smoke if they were from England (vs. Canada and US), former, experimental or current (vs. never) smokers, experimental (vs. never) vapers, age 19 (vs. 16), other/mixed ethnicity, students (vs. don’t know/refused), monthly alcohol users, or past-30-day marijuana users (Table 2). Respondents also had greater odds of reporting that their friends smoke if they were from Canada vs. US (AOR = 1.32 [95% CI = 1.15–1.51], p < .001) or were current vs. experimental smokers (AOR = 3.10 [2.22–4.31], p < .001); there was little evidence for any other differences by smoking or vaping status (all p ≥ .055).

#### 3.3.2 Friend vaping

Respondents had greater odds of reporting that their friends vape if they were from Canada (vs. England), experimental (vs. never) smokers, former, experimental or current (vs. never) vapers, age 17–19, other/mixed ethnicity, monthly alcohol users, or past-30-day marijuana users (Table 2). Respondents also had greater odds of reporting that their friends vape if they were current vs. experimental vapers (AOR = 4.30 [1.71–10.80], p = .002); there was little evidence for any other differences by country, smoking or vaping status (all p ≥ .126).

Post-hoc analyses were used to explore which covariates contributed to higher adjusted odds of friend vaping in Canada compared with England, despite little difference in raw prevalence (Table 2). Unadjusted logistic regressions (data not shown) found little evidence for country differences in friend vaping (all p ≥ .103). Excluding smoking and alcohol use from the fully-adjusted model attenuated the difference between Canada and England (all p ≥ .057). Exclusion of other variables did not influence interpretation of results.

#### 3.3.3 Peer approval of smoking

Respondents had greater odds of perceiving that their peers approve of smoking if they were from England (vs. Canada and US), experimental or current (vs. never) smokers, experimental or current (vs. never) vapers, 16 (vs. 18), female, other/mixed ethnicity, did not know or refused to state their monthly alcohol use, were past-30-day marijuana users, or had 0–2 computers in their household (Table 3). There was little evidence for any other differences between Canada and US or by smoking or vaping status (all p ≥ .162).

#### 3.3.4 Peer approval of vaping

Respondents had greater odds of perceiving that their peers approve of vaping if they were from Canada or US (vs. England), never (vs. experimental and current) smokers, former, experimental or current (vs. never) vapers, female, other/mixed ethnicity, or had 0–1 bathrooms in their household (Table 3). Respondents also had greater odds of perceiving that their peers approve of vaping if they were experimental vs. current smokers (AOR = 1.33 [1.05–1.69], p = .017) or were current vs. experimental vapers (AOR = 1.86 [1.23–2.80], p = .003). There was little evidence for any other differences between Canada and US or by smoking or vaping status (all p ≥ .080).

### 3.4 Interactions between country and smoking and vaping status

#### 3.4.1 Friend smoking

There was little evidence of an interaction between country and smoking (F (4, 10276) = 2.37, p = .0502) or vaping (F (4, 10276) = 2.04, p = .086) status.

#### 3.4.2 Friend vaping

**Fig. 1:**
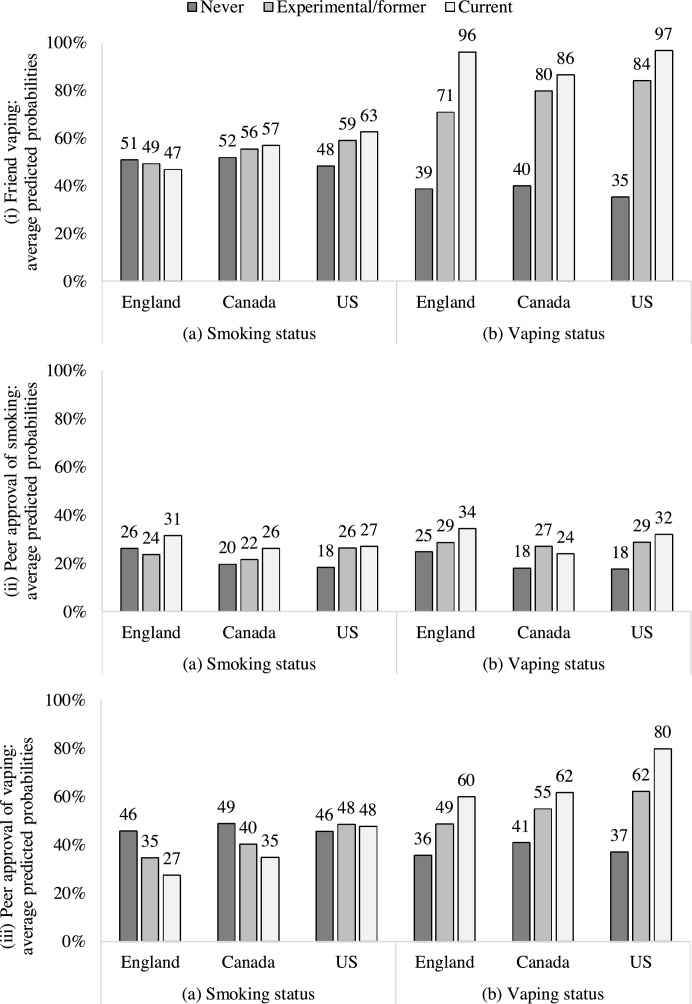
Interactions between country (England, Canada, US) and (a) smoking status (never, experimental/former, current), (b) vaping status (never, experimental/former, current) for (i) friend vaping, (ii) peer approval of smoking, and (iii) peer approval of vaping. Predicted probabilities are weighted and adjusted, n = 10,280.

There was an interaction between country and smoking status (F (4,10276) = 6.27, p < .001; Fig. 1 (i) (a)). In US, friend vaping was higher among experimental/former (AOR = 1.11 [1.07–1.16], p < .001) and current (AOR = 1.15 [1.04–1.28], p = .008) vs. never smokers. There was little evidence of any other differences (all p ≥ .176).

There was also an interaction between country and vaping status (F (4,10276) = 9.64, p < .001; Fig. 1 (i) (b)). In England and US, friend vaping was highest among current vapers (vs. experimental/former: England: AOR = 1.29 [1.19–1.39], p < .001; US: AOR = 1.13 [1.07–1.20], p < .001; vs. never: England: AOR = 1.78 [1.66–1.91], p < .001; US: AOR = 1.85 [1.75–1.95], p < .001) followed by experimental/former vapers (vs. never: England: AOR = 1.38 [1.32–1.45], p < .001; US: AOR = 1.63 [1.57–1.69], p < .001). In Canada, friend vaping was higher among experimental/former (AOR = 1.49 [1.42–1.56], p < .001) and current (AOR = 1.59 [1.35–1.87], p < .001) vs. never vapers only (experimental/former vs. current p = .422).

#### 3.4.3 Peer approval of smoking

There was an interaction between country and smoking status (F (4,10276) = 4.74, p = .001; Fig. 1 (ii) (a)). In England, peer approval of smoking was higher among current vs. experimental/former smokers (AOR = 1.08 [1.00–1.17], p = .044). In US, peer approval of smoking was higher among both experimental/former (AOR = 1.08 [1.04–1.13], p < .001) and current (AOR = 1.09 [1.00–1.19], p = .042) vs. never smokers. There was little evidence of any other differences (all p ≥ .132).

There was some evidence for an interaction between country and vaping status (F (4,10270) = 2.40, p = .048; Fig. 1 (ii) (b)). In Canada, peer approval of smoking was higher among experimental/former vs. never vapers (AOR = 1.09 [1.04–1.15], p < .001). In US, peer approval of smoking was higher among experimental/former (AOR = 1.12 [1.07–1.16], p < .001) and current (AOR = 1.15 [1.02–1.31], p = .026) vs. never vapers. There was little evidence of any other differences (all p ≥ .075).

#### 3.4.4 Peer approval of vaping

There was an interaction between country and smoking status (F (4,10276) = 8.60, p < .001; Fig. 1 (iii) (a)). In England, peer approval of vaping was lowest among current smokers (vs. experimental/former: AOR = 0.93 [0.87–1.00], p = .045; vs. never: AOR = 0.83 [0.78-0.89], p < .001) followed by experimental/former smokers (vs. never: AOR = 0.90 [0.86-0.93], p < .001). In Canada, peer approval of vaping was lower among current (AOR = 0.87 [0.79-0.95], p = .002) and experimental/former (AOR = 0.92 [0.87-0.97], p = .002) vs. never smokers. There was little evidence of any other differences (all p ≥ .201).

There was also an interaction between country and vaping status (F (4,10276) = 5.29, p < .001; Figure (iii) (b)). In England and Canada, peer approval of vaping was higher among current (England: AOR = 1.27 [1.06–1.52], p = .009; Canada: AOR = 1.24 [1.04–1.47], p = .016) and experimental/former (England: AOR = 1.13 [1.08–1.19], p < .001; Canada: AOR = 1.16 [1.10–1.22], p < .001) vs. never vapers. In US, peer approval of vaping was highest among current vapers (vs. experimental/former: AOR = 1.20 [1.09–1.32], p < .001; vs. never: AOR = 1.53 [1.39–1.69], p < .001) followed by experimental/former vapers (vs. never: AOR = 1.28 [1.22–1.34], p < .001).

## 4 Discussion

This study was the first to our knowledge to assess country differences in social norms towards smoking and vaping among youth and associations with product use. Overall, youth had more pro-vaping than pro-smoking norms. English youth reported the most pro-smoking and least pro-vaping norms overall. Norms were similar in Canada and the US, except more Canadian than US youth reported friend smoking. Smokers had more pro-smoking norms, vapers had more pro-vaping norms, and there were some cross-product associations between norms and use.

Prevalence of friend use of either product was similar overall, with around half of youth reporting friend smoking (47%) and friend vaping (52%), while perceived peer approval of vaping (44%) was almost twice that of peer approval of smoking (23%). Friend smoking and vaping was greater than previous British (East et al., 2018) and US (Barrington-Trimis et al., 2016; Gorukanti et al., 2017) studies, possibly due to the older age of participants in this study. To our knowledge, perceived peer approval of smoking and vaping have not been simultaneously assessed in other studies. The finding that peer approval of vaping was greater than smoking is perhaps unsurprising given that vaping is less harmful than smoking (McNeill et al., 2015) and may be more appealing to youth than smoking given the novelty and range of products and flavors available.

The high prevalence of friend product use and perceived peer approval of vaping is concerning, particularly given the age of respondents, that the majority had never smoked or vaped, and that norms were associated with use. There are concerns that the popularity of vaping may lead never smoking youth to try nicotine (US Department of Health and Human Services, 2016). Between 2017 and 2018, prevalence of vaping increased in Canada and the US, including among never smokers, but did not change in England (Hammond et al., 2019); this mirrors our findings that vaping norms were most positive in the former two countries. It is important that norms are continuously monitored alongside prevalence to explore whether pro-vaping norms could precede any changes in vaping, or smoking.

Friend smoking and peer approval of smoking were more commonly reported by smokers, adding to the large body of evidence that smoking norms influence smoking behavior (Chang et al., 2006; Chapman and Freeman, 2008; Conner et al., 2017; Lotrean et al., 2013; Van De Ven et al., 2007). Friend vaping and peer approval of vaping were more commonly reported by vapers, also similar to previous studies (Gorukanti et al., 2017; Lozano et al., 2019; Thrasher et al., 2016). Interestingly, there was a strong dose-response association between greater vaping behavior and pro-vaping norms in all countries which was not mirrored for smoking.

Considering cross-product norm-behavior associations, friend smoking, friend vaping, and peer approval of smoking were more common among smokers and vapers, while peer approval of vaping was more common among vapers but less common among smokers. This suggests that associations between norms and behavior may not be product-specific, although generally product-specific associations were stronger than cross-product associations, particularly for friend smoking and vaping. Except the association between vaping and friend smoking (Lozano et al., 2019; Morello et al., 2016; Thrasher et al., 2016), the direction of associations are inconsistent with previous studies (East et al., 2018; Lozano et al., 2019). This, combined with the mixed positive and negative associations between norms towards one product and use of the alternative, precludes any firm conclusions regarding the potential of e-cigarettes to renormalize smoking. Further research assessing specific norm-behavior pathways using longitudinal methodology is needed.

Both friend smoking and vaping were more common among youth who used alcohol monthly and were older, although increases in friend vaping emerged earlier than friend smoking. Both peer approval of smoking and vaping were more common among females, and generally all norms were more positive among marijuana users (except peer approval of vaping) and ethnic minorities. Thus, there may be some shared risk factors for holding positive smoking and vaping norms.

The country differences in smoking and vaping norms were surprising. English youth reported more pro-smoking norms than the US despite similar smoking rates (Hammond et al., 2019) and the US having less comprehensive tobacco control policies (ITC, 2018; WHO, 2017). Moreover, friend smoking was higher in Canada than the US, yet peer approval of smoking was similar, contrary to Canada’s lower smoking prevalence (Hammond et al., 2019; WHO, 2015) and more comprehensive tobacco control policies (ITC, 2018; WHO, 2017). However, the finding that English youth reported more pro-smoking norms than Canadian youth does align with prevalence rates (Hammond et al., 2019; WHO, 2015), Canada’s longer history of tobacco control policies (Hammond et al., 2006; ITC, 2018; WHO, 2017) and a recent study among adult daily smokers (East et al., 2019b).

Canada and the US had more pro-vaping norms than England in adjusted analyses, while Canada and the US did not differ. However, at the time of surveying, Canada had the lowest vaping prevalence among this age group (Hammond et al., 2019) and the most restrictive vaping policies (Gravely et al., 2019; ITC, 2018). These results may suggest that vaping policies or prevalence rates may have less influence on social norms than some might suppose, reflecting findings from a recent study in Europe (East et al., 2019a) yet contrary to findings among adult smokers and ex-smokers in England, Canada, the US, and Australia (Aleyan et al., 2019).

The discrepancy between the unadjusted and adjusted country differences in friend vaping warrants further exploration. Despite the adjusted odds of friend vaping being higher in Canada than England, exclusion of smoking and alcohol use attenuated country differences. However, inclusion of alcohol, and marijuana, use is important in studies assessing youth smoking and vaping, since they may serve as a proxy for risky behavior that confounds apparent associations (Kozlowski and Warner, 2017).

It is important to mention that while tobacco control policies in 2017 were generally least comprehensive in the US, and vaping policies most restrictive in Canada, the detailed picture is more complicated. Canada’s vaping restrictions were generally unenforced (Hammond et al., 2015) and after survey administration Canada implemented a new Vaping Products Act which relaxed many restrictions (Health Canada, 2018). Further, some youth also use vaping devices for marijuana or cannabis oil (Cassidy et al., 2018; Thurtle et al., 2017) and around the time of survey administration recreational marijuana use was legalized in Canada and several US states. Both the new Act and marijuana legalization were widely discussed around the time of this survey and may have influenced vaping norms; however, this is only speculation and would be difficult to assess. In addition to national smoking/vaping policies, Canada and the US also have divergent municipal/state/province-level policies (e.g., smoke free legislation, minimum age for legal purchase of nicotine products (US: 18–21; Canada: 18–19)). Moreover, since these data were collected in 2017, vaping environments have changed in Canada and the US: while Canada has relaxed many restrictions (Health Canada, 2018), the US has launched a national youth vaping prevention campaign (Food and Drug Administration, 2019) and proposed banning vaping in some jurisdictions (City Attorney of San Francisco, 2019). Youth vaping prevalence has also increased in these two countries since 2017 (Hammond et al., 2019). Therefore, while this study provides an overview of norms in each country, they may differ at the municipal/state/province-level and may not be generalizable to more recent years.

The findings from this study must be considered in light of several limitations. First, data were cross-sectional, meaning that directionality cannot be inferred, and there is almost certainly a reciprocal relationship between norms and behavior. Second, smoking norms may confound vaping norms and vice-versa; for example, some youth could have pro-vaping norms *because* they have anti-smoking norms. Future research could assess within-person differences in smoking/vaping norms and their associations with product use. Third, item wording for friend use does not enable differentiation between those with only one smoking/vaping friend and those among whom all friends smoke/vape. Fourth, data were self-reported and may be subject to recall and social desirability biases, which may be particularly pronounced when asking about peer’s nicotine use and norms. However, the fact that this was an anonymous, self-administered survey may alleviate some of this concern. Fifth, as mentioned above, Canada and the US have differing policies at the municipal/state/province-level which were not accounted for in analyses.

Despite these limitations, this study has important strengths. First, although participants were recruited from non-probability-based commercial samples, sample weights were incorporated to enhance representativeness and both weighted and unweighted estimates were similar to national benchmark surveys in each country (Hammond et al., 2019). Second, the sample was large, allowing for assessment of different user status groups and interactions with country. Third, this study is the first to compare social norms towards smoking and vaping and their associations with product use across countries, providing a novel contribution to the literature.

## 5 Conclusions

Around half of youth reported having friends who smoke and vape. Perceived peer approval of vaping (44%) was twice that of peer approval of smoking (23%). English youth had the most pro-smoking norms but least pro-vaping norms overall, contrary to regulatory environments. Consistent with previous research, smokers reported more pro-smoking norms while vapers reported more pro-vaping norms. There were also cross-product associations between norms and behavior, although product-specific associations were stronger than cross-product associations.

## Role of funding source

This project has been made possible through a P01 grant (1P01CA200512-01) from the US National Institutes of Health. Additional support was provided by a Canadian Institutes of Health Research (CIHR)-Public Health Agency of Canada (PHAC) Applied Public Health Research Chair (David Hammond) and US National Institutes of Health R01 grant (R01 TW010652; James F Thrasher). Katherine East’s PhD is funded by the UK Centre for Tobacco and Alcohol studies (MR/K023195/1). The UK Public Health Research Consortium funded the development of the social norms measures included in this study.

## Contributors

Katherine East led the data analysis and write-up of the manuscript.

Sara Hitchman and Ann McNeill provided input on the research questions, survey design, analysis plan, interpretation of results, manuscript write-up, and critically reviewing the manuscript.

James Thrasher provided input on the survey design, analysis plan, interpretation of the results, and critically reviewing the manuscript.

David Hammond was the Principal Investigator of the Youth Tobacco and Vaping Survey used in this study, and provided input on the survey design, analysis plan, interpretation of the results, and critically reviewing the manuscript.

All authors approved the final manuscript as submitted and agree to be accountable for all aspects of the work.

## Declaration of Competing Interest

James Thrasher and David Hammond have served on behalf of governments in response to legal challenges from the tobacco industry. All other authors have no conflicts of interest.
